# CBC-Derived Inflammatory Indices and Myocardial Injury Severity at Presentation in Acute Myocardial Infarction: Association and Discriminative Performance

**DOI:** 10.3390/jcm15114397

**Published:** 2026-06-05

**Authors:** Putrada Ninla-aesong, Sasithorn Sanakus, Chennet Phonphet, Jom Suwanno, Ladda Thiamwong

**Affiliations:** 1 School of Medicine and Research Center in Tropical Pathobiology, Walailak University, Nakhon Si Thammarat 80160, Thailand; 2 Maharaj Nakhon Si Thammarat Hospital, Nakhon Si Thammarat 80000, Thailand; junioryuita@gmail.com; 3 School of Nursing and the Excellent Center of Community Health Promotion, Walailak University, Nakhon Si Thammarat 80160, Thailand; pchennet@wu.ac.th (C.P.); sjom@wu.ac.th (J.S.); 4 College of Nursing, University of Central Florida, Orlando, FL 32827, USA; ladda.thiamwong@ucf.edu

**Keywords:** acute myocardial infarction (AMI), ST-segment elevation myocardial infarction (STEMI), non-ST-segment elevation myocardial infarction (NSTEMI), myocardial injury severity, high-sensitivity Troponin T (hs-Troponin T), neutrophil-to-lymphocyte ratio (NLR), neutrophil-to-lymphocyte × platelet ratio (NLPR), systemic inflammatory indices, early diagnosis, biomarkers

## Abstract

**Background:** Early assessment of myocardial injury severity at presentation remains challenging in acute myocardial infarction (AMI). Complete blood count (CBC)-derived inflammatory indices may provide accessible adjunctive biomarkers reflecting early systemic inflammatory activation associated with myocardial injury. This study evaluated the association and discriminative performance of CBC-derived inflammatory indices for presentation-time myocardial injury severity. **Methods:** This retrospective study included 252 patients with AMI. CBC-derived inflammatory indices, including the neutrophil-to-lymphocyte ratio (NLR) and neutrophil-to-lymphocyte × platelet ratio (NLPR), were calculated from blood samples obtained at presentation (0 h). Correlation analysis, multivariable linear regression, logistic regression, incremental model analysis, and receiver operating characteristic (ROC) analysis were performed to assess associations with high-sensitivity Troponin T (hs-Troponin T) levels and high myocardial injury, defined as the highest hs-Troponin T tertile. **Results:** Both log NLR and log NLPR showed significant positive correlations with log hs-Troponin T (ρ = 0.422 and 0.396, respectively; *p* < 0.001). In multivariable linear regression adjusted for clinical variables and AMI subtype, log NLR (B = 0.88, *p* < 0.001) and log NLPR (B =0.77, *p* < 0.001) remained independently associated with log hs-Troponin T. Incremental model analysis demonstrated significant increases in explanatory performance after addition of log NLR (ΔR^2^ = 0.137) and log NLPR (ΔR^2^ = 0.121, *p* < 0.001). In logistic regression, log NLR (adjusted OR 2.77, 95% CI 1.65–4.66) and log NLPR (adjusted OR 2.46, 95% CI 1.53–3.95) were independently associated with high myocardial injury. ROC analysis demonstrated modest improvement in discrimination after incorporation of inflammatory indices, with AUC increasing from 0.709 for the baseline clinical model to 0.778 with log NLR and 0.770 with log NLPR. Supplementary reclassification analyses demonstrated improved classification performance. **Conclusions:** CBC-derived inflammatory indices, particularly NLR and NLPR, were independently associated with presentation-time myocardial injury severity in patients with AMI, even after adjustment for AMI subtype. Although improvements in ROC-based discrimination were modest, supplementary reclassification analyses suggested incremental value beyond conventional clinical variables and AMI subtype. These findings support the potential utility of CBC-derived inflammatory indices for early assessment of myocardial injury during AMI presentation.

## 1. Introduction

Acute myocardial infarction (AMI) remains a leading cause of morbidity and mortality worldwide despite advances in early diagnosis and reperfusion therapy. According to the Fourth Universal Definition of Myocardial Infarction, AMI is classified into ST-segment elevation myocardial infarction (STEMI) and non-ST-segment elevation myocardial infarction (NSTEMI), based on electrocardiographic findings and evidence of myocardial injury reflected by cardiac Troponin elevation [1].

STEMI is typically associated with complete coronary occlusion and larger infarct size, whereas NSTEMI often results from partial occlusion and variable degrees of myocardial injury [2]. Because the extent of myocardial injury is a key determinant of clinical outcomes, including left ventricular dysfunction, heart failure, and mortality [3,4], early assessment of myocardial injury severity at presentation may provide important prognostic information in patients with AMI.

High-sensitivity Troponin T (hs-Troponin T) reflects myocardial injury across both STEMI and NSTEMI, correlates with myocardial injury severity, and is widely used as the gold-standard biomarker for myocardial injury [4]. However, hs-Troponin T elevation may be delayed during the early phase of myocardial injury. At early presentation (0–1 h) or within the initial hours after symptom onset, hs-Troponin T levels may remain normal or only mildly elevated despite ongoing myocardial ischemia and evolving injury, limiting its sensitivity at presentation [4,5,6]. However, because inflammatory activation occurs early in the pathophysiological cascade of AMI [7], complete blood count (CBC)-derived inflammatory indices may provide complementary information reflecting early systemic inflammatory responses associated with myocardial injury.

Furthermore, circulating cardiac Troponin levels may be influenced by factors other than myocardial infarction, including age, sex, renal dysfunction, time from chest pain onset, critically illness, and chronic subclinical myocardial injury, which may complicate interpretation of absolute values in certain clinical contexts [4,6]. In addition, access to Troponin testing may be limited in some healthcare settings. These limitations support the potential value of additional biomarkers that provide complementary information regarding early biological processes associated with myocardial injury.

Systemic inflammation plays a central role in the pathophysiology of AMI. Inflammatory responses contribute to plaque instability, thrombus formation, microvascular dysfunction, and subsequent myocardial injury and necrosis [7,8,9]. Elevated leukocyte and neutrophil counts are associated with larger infarct size, impaired left ventricular function, and adverse clinical outcomes following myocardial infarction [10,11,12,13,14]. Platelets further amplify thrombo-inflammatory responses through aggregation and interaction with leukocytes [15]. These findings support the biological plausibility of inflammatory biomarkers for assessing myocardial injury severity.

Inflammatory responses occur early in the pathophysiological cascade and may precede detectable Troponin elevation. CBC-derived inflammatory indices, including the neutrophil-to-lymphocyte ratio (NLR), monocyte-to-lymphocyte ratio (MLR), and platelet-to-lymphocyte ratio (PLR), have gained increasing attention as accessible markers of systemic inflammation. Among these, NLR has been most extensively studied and consistently associated with adverse cardiovascular outcomes, including increased risk of complications and mortality in patients with acute coronary syndromes (ACS) and myocardial infarction [14,16,17,18,19,20,21,22].

In addition, NLR has demonstrated prognostic value in multiple ACS populations and has been reported to outperform MLR and PLR in predicting adverse outcomes [23,24,25]. Furthermore, NLR has been suggested to be associated with increases in Troponin levels in patients with non-ST-segment elevation acute coronary syndrome (NSTE-ACS) in emergency settings [22]. More recently, in addition to PLR, the neutrophil-to-lymphocyte × platelet ratio (NLPR), a novel platelet-related inflammatory index, has been proposed as an accessible marker of systemic inflammation and has been associated with a higher risk of major adverse cardiovascular events (MACE) in patients with ACS undergoing percutaneous coronary intervention (PCI) [26].

In our prior analysis of patients presenting with AMI undergoing PCI, multiple CBC-derived inflammatory indices were evaluated for early identification of STEMI at first medical contact. Among these, NLPR, which may capture aspects of the interplay between inflammation and thrombosis, demonstrated the highest sensitivity and negative predictive value, supporting its potential role as an early screening marker, whereas NLR showed moderate but consistent diagnostic performance across analyses. These findings suggested that NLR and NLPR may provide complementary information to support early identification of STEMI in emergency settings [27]. However, while our previous study focused on diagnostic performance for early STEMI identification, their relationship with myocardial injury severity has not been fully elucidated. In particular, it is unclear whether indices such as NLR and NLPR reflect the extent of myocardial injury at presentation, as indicated by high-sensitivity troponin levels. Furthermore, their relative performance in relation to myocardial injury severity, assessed using multiple complementary analytical approaches within the same cohort, remains incompletely defined.

Composite indices derived from routine CBC parameters, including NLR and NLPR, may provide integrative measures of systemic inflammatory and thrombotic activity. Clarifying their association with myocardial injury severity and their incremental value beyond conventional clinical factors may improve understanding of the role of inflammation in myocardial injury and support the potential utility of these indices as adjunctive biomarkers.

Therefore, the present study focused on NLR and NLPR to evaluate their association with Troponin-based myocardial injury severity at presentation in patients with AMI. Because inflammatory activation occurs early in the pathophysiological cascade of AMI, CBC-derived inflammatory indices may provide complementary information regarding systemic inflammatory responses associated with myocardial injury. In addition to evaluating their associations with myocardial injury severity, we further assessed their incremental explanatory, discriminative, and reclassification value beyond conventional clinical variables using multiple complementary analytical approaches.

## 2. Materials and Methods

### 2.1. Study Design and Setting

This retrospective, cross-sectional diagnostic study was conducted at Maharaj Nakhon Si Thammarat Hospital, a tertiary referral center for cardiovascular care in Nakhon Si Thammarat, Thailand. The study was performed in accordance with the principles of the Declaration of Helsinki and was approved by the Ethics Committee of Walailak University, Thailand (protocol code: WUEC-25-179-01 and date of approval: 28 May 2025). Due to the retrospective design and use of de-identified data, the requirement for informed consent was waived.

### 2.2. Study Population and Sample Selection

The study population comprised consecutive patients diagnosed with AMI, including STEMI and NSTEMI, who underwent PCI during their index hospitalization between 1 October 2020 and 30 September 2021. Clinical, laboratory, and procedural data were retrospectively obtained from electronic medical records and hospital databases.

Because the present study focused on Troponin-based myocardial injury severity within patients with confirmed AMI, patients diagnosed with unstable angina were excluded to ensure a homogeneous population with confirmed myocardial injury. After applying the eligibility criteria, a total of 252 patients with AMI were included in the final analysis, comprising 195 patients with STEMI and 57 patients with NSTEMI.

### 2.3. Diagnosis and Classification of Acute Myocardial Infarction

Patients were initially evaluated for suspected ACS at first medical contact. The final diagnosis of AMI, including STEMI and NSTEMI, was confirmed by a board-certified cardiologist based on clinical presentation, electrocardiographic findings, serial hs-Troponin T measurements demonstrating a rise and/or fall pattern, and coronary angiographic findings, in accordance with the Fourth Universal Definition of Myocardial Infarction [1] and the 2020 European Society of Cardiology guidelines [4].

The Fourth Universal Definition (2018) [1] defines acute myocardial injury as a rise/fall of cardiac Troponin levels with at least one value above the 99th percentile upper reference limit (URL). Myocardial infarction is diagnosed when acute myocardial injury occurs in the presence of clinical evidence of myocardial ischemia, including myocardial ischemic symptoms, ECG changes, pathological Q waves, imaging evidence, or coronary thrombus [1].

STEMI was identified based on persistent ST-segment elevation on a 12-lead electrocardiogram (ECG), whereas NSTEMI was defined by the absence of persistent ST-segment elevation [1,4].

Patients diagnosed with unstable angina were excluded because the present study focused on Troponin-based myocardial injury severity in patients with AMI.

### 2.4. Inclusion Criteria and Exclusion Criteria

#### 2.4.1. Inclusion Criteria

Patients were included if they had a final diagnosis of AMI, including STEMI or NSTEMI, and underwent PCI during the index hospitalization. Eligible patients were required to have complete clinical records and laboratory data available for analysis. In addition, baseline diagnostic data obtained at presentation (0 h) were required, including a 12-lead electrocardiogram (ECG), hs-Troponin T, and CBC with differential. These baseline values were used for subsequent analysis of inflammatory indices and assessment of Troponin-based myocardial injury severity.

#### 2.4.2. Exclusion Criteria

Patients diagnosed with unstable angina were excluded because the present study specifically focused on Troponin-based myocardial injury severity in patients with AMI. Additional exclusion criteria included known hematologic malignancy or bone marrow disease, receipt of immunosuppressive therapy, active infection or sepsis, and a history of chronic inflammatory or autoimmune disease, as these conditions may significantly affect systemic inflammatory markers. Patients who had undergone major surgery or experienced significant trauma within 30 days before hospitalization were also excluded. Furthermore, patients with incomplete clinical records or missing laboratory data required for analysis were excluded.

**Figure 1:**
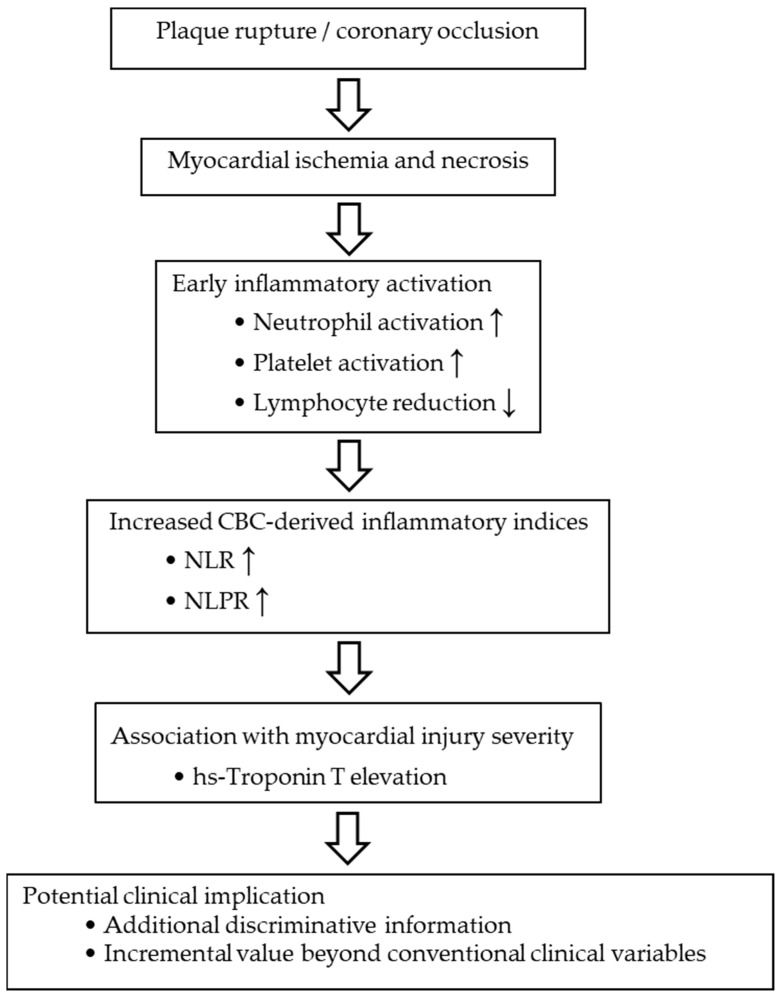
Proposed relationship between inflammatory activation and myocardial injury severity during acute myocardial infarction presentation. Schematic illustration of the proposed relationship between myocardial ischemia, inflammatory activation, neutrophil and platelet responses, CBC-derived inflammatory indices (NLR and NLPR), hs-Troponin T elevation, and their potential incremental and discriminative value during AMI presentation. Abbreviations: CBC, complete blood count; NLR, neutrophil-to-lymphocyte ratio; NLPR, neutrophil-to-lymphocyte–platelet ratio; ↑, increase; ↓ decrease.

The study population, inclusion criteria, exclusion criteria, and final sample selection process are summarized in Figure 1. Patient screening and sample selection were performed using criteria similar to those described in our previous study of the same AMI cohort [27].

### 2.5. Data Collection

Demographic characteristics, cardiovascular risk factors, clinical presentation, 12-lead electrocardiographic findings, hs-Troponin T measurements, coronary angiographic findings, and laboratory data obtained at presentation (0 h), as well as serial measurements obtained during hospitalization, were retrospectively extracted from patient medical records.

For the primary analysis, baseline measurements obtained at presentation (0 h) were used to ensure temporal consistency across variables and to reflect the early biological state at presentation and minimize bias related to disease progression or treatment effects. Although serial assessments were performed as part of routine clinical care, only baseline values were used for calculation of inflammatory indices and covariate adjustment.

Baseline demographic and clinical variables included age, sex, smoking status, hypertension, diabetes mellitus, dyslipidemia, renal function, and AMI subtype. Additional laboratory variables included lipid profiles and estimated glomerular filtration rate (eGFR). These variables were included as covariates based on biological plausibility and prior evidence suggesting potential associations with systemic inflammatory responses and myocardial injury severity. In particular, renal function was included because impaired renal function may influence circulating hs-Troponin T levels and systemic inflammatory markers independent of acute myocardial injury [4]. AMI subtype was included because STEMI and NSTEMI may differ in infarct burden, Troponin kinetics, and inflammatory activation.

Laboratory measurements included CBC with differential, hs-Troponin T, lipid profiles, renal function tests, and eGFR, all performed using routine commercial assays according to standard operating procedures. Renal function was assessed using eGFR, calculated from serum creatinine values.

### 2.6. CBC-Derived Inflammatory Indices

Systemic inflammatory indices were calculated based on baseline CBC data obtained at presentation (0 h). Baseline values were used to reflect the early inflammatory response at presentation and to minimize potential bias related to treatment effects or disease progression.

In the present study, the NLR and NLPR were selected as the primary CBC-derived inflammatory indices of interest based on their biological relevance and prior findings demonstrating their potential diagnostic value in AMI. These indices were calculated using baseline CBC values obtained at presentation.

The indices were calculated using the following formulas:

- NLR = Neutrophil count/Lymphocyte count.
- NLPR = Neutrophil count/(Lymphocyte count × Platelet count).

NLR reflects the balance between innate inflammatory activation, represented by neutrophils, and adaptive immune regulation, represented by lymphocytes. NLPR incorporates platelet count to account for the interaction between inflammation and thrombosis, which plays a critical role in the pathophysiology of AMI.

### 2.7. Outcome Definition

The primary outcome was myocardial injury severity assessed by hs-Troponin T levels obtained at presentation (0 h). Baseline hs-Troponin T values were used to reflect early myocardial injury at presentation and to maintain temporal consistency with CBC-derived inflammatory indices measured at the same time point. Presentation-time hs-Troponin T was selected to maintain temporal consistency with baseline CBC-derived inflammatory indices and to reflect early myocardial injury during AMI presentation.

Because all included patients had confirmed AMI, hs-Troponin T values were used to quantify the extent of myocardial injury rather than for diagnostic classification. This approach allowed evaluation of CBC-derived inflammatory indices in relation to injury severity within a relatively homogeneous population with confirmed myocardial injury.

hs-Troponin T was analyzed as a continuous variable to preserve quantitative information regarding the extent of myocardial injury. Because hs-Troponin T and inflammatory indices demonstrated right-skewed distributions, logarithmic transformation was applied prior to analysis to approximate normality, reduce the influence of extreme values, and satisfy assumptions required for regression analyses.

For secondary analyses, hs-Troponin T values were categorized into tertiles. Because no universally accepted clinical threshold exists for defining myocardial injury severity using presentation-time hs-Troponin T levels, patients in the highest tertile were classified as having high myocardial injury for discriminative analyses. The lower two tertiles were categorized as lower myocardial injury severity. This approach allowed data-driven stratification while maintaining adequate sample size within subgroups for statistical analyses.

### 2.8. Statistical Analysis

Continuous variables were expressed as median and interquartile range (IQR), while categorical variables were presented as frequencies and percentages. Because hs-Troponin T and inflammatory indices demonstrated right-skewed distributions, logarithmic transformation was applied prior to correlation and regression analyses. All statistical tests were two-tailed, and a *p*-value < 0.05 was considered statistically significant. Statistical analyses were performed using SPSS software (version 22, Chicago, IL, USA) and MedCalc Statistical software (version 23.5.1; MedCalc Software Ltd., Ostend, Belgium). Continuous net reclassification improvement (NRI) and integrated discrimination improvement (IDI) analyses were performed using Python-based statistical computation (Python Software Foundation, version 3.13).

#### 2.8.1. Correlation Analysis

Spearman’s rank correlation analysis was performed to evaluate associations between log-transformed inflammatory indices and log-transformed hs-Troponin T levels. This non-parametric method was selected because it does not assume normal distribution or linear relationships and is less sensitive to outliers. Correlation results were reported as correlation coefficients (ρ) with corresponding two-tailed *p*-values. Scatter plots using log-transformed values were constructed to visually assess relationships between inflammatory indices and hs-Troponin T.

#### 2.8.2. Multivariable Linear Regression Analysis

Multivariable linear regression analysis was performed to evaluate independent associations between CBC-derived inflammatory indices and myocardial injury severity using log-transformed hs-Troponin T as the dependent variable. Clinical covariates were selected a priori based on biological plausibility and prior evidence, including age, sex, smoking status, hypertension, diabetes mellitus, dyslipidemia, eGFR, and AMI subtype. AMI subtype (STEMI/NSTEMI) was included because of potential differences in infarct burden, Troponin kinetics, and inflammatory activation between AMI subtypes. All covariates were included regardless of statistical significance to control for potential confounding. Time from symptom onset to presentation was not included due to potential inaccuracy and inconsistency in patient-reported symptom onset, which may not reliably reflect the true onset of myocardial injury.

Separate regression models were constructed for each inflammatory index to avoid multicollinearity. Results were presented as unstandardized regression coefficients (B) with 95% confidence intervals (CI).

#### 2.8.3. Incremental Value Analysis (ΔR^2^)

To determine whether CBC-derived inflammatory indices provided additional explanatory value beyond the predefined clinical model, hierarchical multivariable linear regression analysis was performed. A baseline model (Model 1) included predefined clinical covariates. Each inflammatory index (log NLR or log NLPR) was then added individually to construct extended models (Model 2).

Incremental value was quantified as the change in the coefficient of determination (ΔR^2^) between nested models, representing the additional proportion of variance explained by inclusion of the inflammatory index. Statistical significance of ΔR^2^ was assessed using the F-change test.

#### 2.8.4. Logistic Regression and Discriminative Performance Analysis

Multivariable logistic regression analysis was performed using the same predefined clinical model to evaluate factors associated with high myocardial injury, defined as the highest hs-Troponin T tertile. Predicted probabilities derived from logistic regression models were used to construct receiver operating characteristic (ROC) curves.

Model discrimination was assessed using the area under the ROC curve (AUC) with 95% confidence intervals. Differences in AUC (ΔAUC) between baseline and extended models were assessed using DeLong’s test for correlated ROC curves. In addition, continuous NRI and IDI analyses were performed to assess incremental classification performance after incorporation of inflammatory indices.

## 3. Results

### 3.1. Baseline Characteristics

Table 1 presents the baseline demographic, clinical, and laboratory characteristics of the study population. A total of 252 patients with AMI were included, comprising 195 (77.4%) with STEMI and 57 (22.6%) with NSTEMI.

The median age was 64 years (interquartile range [IQR], 15), and 73% of participants were male. Smoking was reported in 39.3% of patients. The most common comorbidities were hypertension (39.7%), dyslipidemia (28.2%), and diabetes mellitus (21.4%). The mean body mass index (BMI) was 23.44 ± 4.05 kg/m^2^. The mean eGFR was 78.19 ± 24.24 mL/min/1.73 m^2^, indicating generally preserved renal function.

At presentation, the median hs-Troponin T level was 416.00 ng/L (IQR, 1718.75), with a wide distribution reflecting heterogeneous myocardial injury severity. The median neutrophil, lymphocyte, and platelet counts were 7568 (IQR, 5561), 1638 (IQR, 1160), and 242.00 (IQR, 89.50), respectively. The median NLR was 4.47 (IQR, 5.13), and the median NLPR was 1074.00 (IQR, 1210.00).

### 3.2. Correlation Between Inflammatory Indices and Myocardial Injury

**Figure 2:**
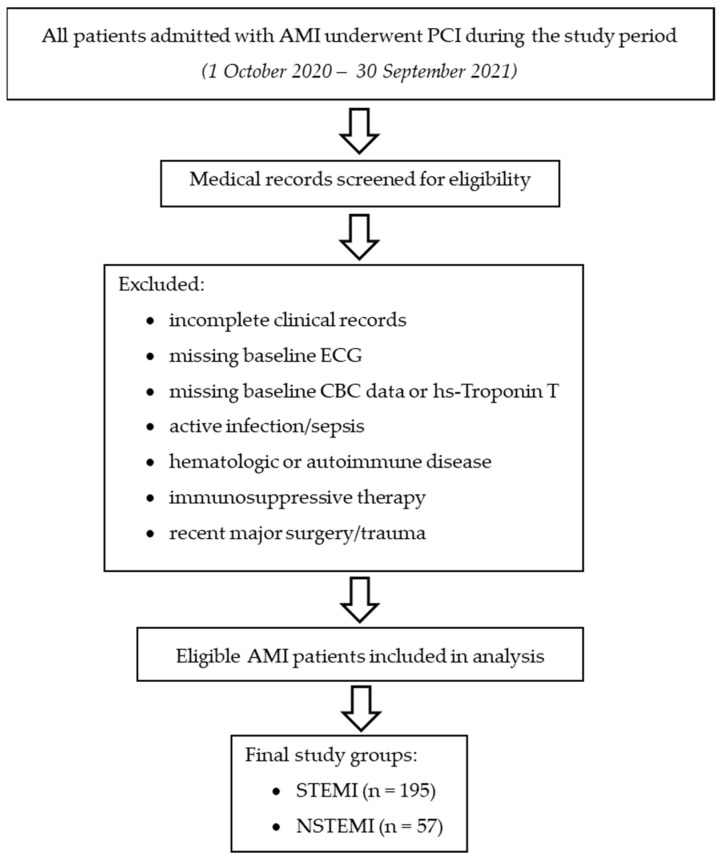
Flow chart of the study population and sample selection. Abbreviations: STEMI, ST-segment elevation myocardial infarction; NSTEMI, non-ST-segment elevation myocardial infarction.

**Figure 3:**
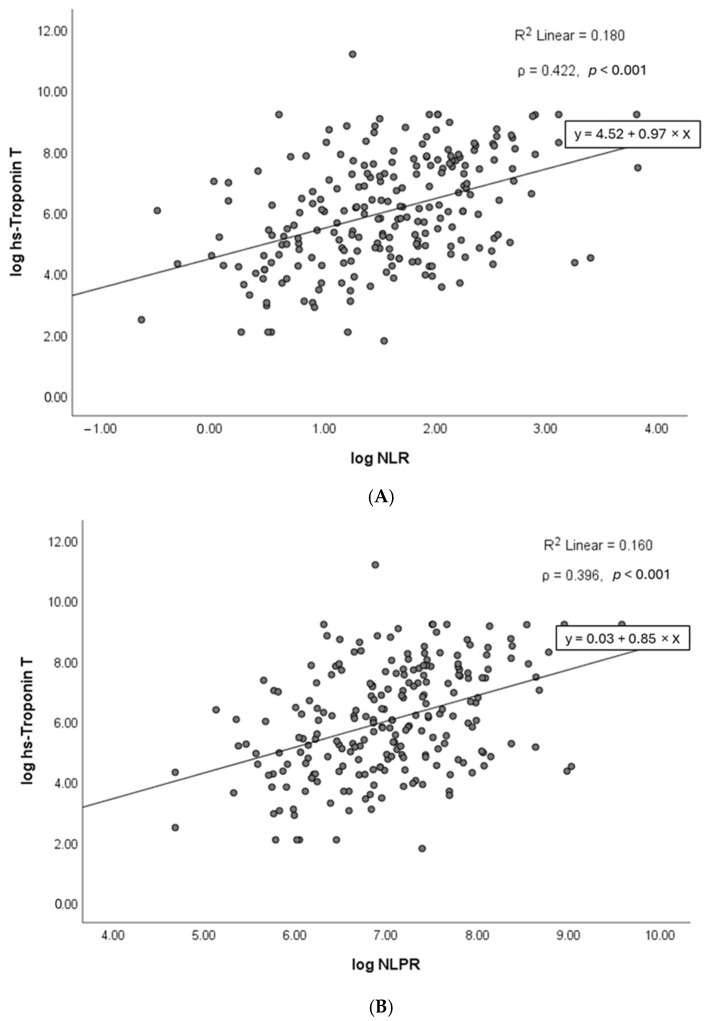
Association between CBC-derived inflammatory indices and myocardial injury severity at presentation (0 h). (**A**) Scatter plot illustrating the relationship between log-transformed neutrophil-to-lymphocyte ratio (log NLR) and log-transformed hs-Troponin T (log hs-Troponin T). (**B**) Scatter plot illustrating the relationship between log-transformed neutrophil-to-lymphocyte × platelet ratio (log NLPR) and log-transformed hs-Troponin T (log hs-Troponin T). Each point represents an individual patient. Both axes are presented on a logarithmic scale to account for right-skewed distributions of the variables and to better visualize their relationships. Solid lines represent fitted linear regression lines. Spearman’s correlation coefficients (ρ) and corresponding *p*-values are displayed within each panel. Abbreviations: NLR, neutrophil-to-lymphocyte ratio; NLPR, neutrophil-to-lymphocyte–platelet ratio; hs-Troponin T, high-sensitivity Troponin T.

Scatter plot analyses demonstrated positive associations between inflammatory indices and myocardial injury at presentation (Figure 2 and Figure 3). Log-transformed NLR (log NLR) showed a moderate positive relationship with log-transformed hs-Troponin T (R^2^ = 0.180; Figure 3A). while log NLPR demonstrated a similar association (R^2^ = 0.160; Figure 3B).

Consistent with these findings, Spearman correlation analysis revealed significant positive correlations with log hs-Troponin T (Table 2). Log NLR showed the strongest correlation (ρ = 0.422, *p* < 0.001), followed by log NLPR (ρ = 0.396, *p* < 0.001).

### 3.3. Multivariable Regression Analysis

Multivariable linear regression analysis was performed to evaluate independent associations between inflammatory indices and myocardial injury severity using log-transformed hs-Troponin T as the dependent variable (Table 3). In Model 1, which included clinical variables (including age, sex, smoking status, diabetes mellitus, hypertension, dyslipidemia, eGFR, and AMI subtype) only, AMI subtype was significantly associated with log hs-Troponin T, whereas other demographic or clinical factors were not significantly associated with log hs-Troponin T.

After inclusion of inflammatory indices, log NLR (Model 2) remained significantly associated with log hs-Troponin T (B = 0.88, 95% confidence interval (CI): 0.60 to 1.16, *p* < 0.001). Similarly, log NLPR (Model 3) showed a significant association with log hs-Troponin T (B = 0.77, 95% CI: 0.51 to 1.03, *p* < 0.001).

Conventional cardiovascular risk factors, including age, sex, smoking status, diabetes mellitus, hypertension, dyslipidemia, and eGFR, were not significantly associated with log hs-Troponin T in any model.

### 3.4. Incremental Association of Inflammatory Indices with hs-Troponin T Levels

Incremental model analysis demonstrated that the addition of inflammatory indices significantly improved the explanatory performance of log-transformed hs-Troponin T (Table 4). Model 1, which included clinical variables and AMI subtype, showed limited explanatory performance (R^2^ = 0.105).

Adding of log NLR (Model 2) significantly improved model performance, increasing the explained variance to R^2^ = 0.242 (ΔR^2^ = 0.137, *p* < 0.001). Similarly, incorporation of log NLPR (Model 3) significantly improved model performance, increasing the explained variance to R^2^ = 0.226 (ΔR^2^ = 0.121, *p* < 0.001). Model 2 demonstrated slightly greater improvement compared with Model 3.

### 3.5. Multivariable Logistic Regression Analysis for High Myocardial Injury

Multivariable logistic regression analysis was performed to evaluate factors associated with high myocardial injury, defined as the highest tertile of hs-Troponin T at presentation (Table 5). In Model 1, AMI subtype was independently associated with high myocardial injury (adjusted OR 16.99, 95% CI 2.24–129.16, *p* = 0.006), whereas other demographic and clinical variables were not significantly associated with high myocardial injury.

After incorporating inflammatory indices, both log NLR and log NLPR remained independently associated with high myocardial injury. In Model 2, log NLR was significantly associated with high myocardial injury (adjusted OR 2.77, 95% CI 1.65–4.66, *p* < 0.001). Similarly, in Model 3, log NLPR was independently associated with high myocardial injury (adjusted OR 2.46, 95% CI 1.53–3.95, *p* < 0.001).

### 3.6. Discriminative Performance for High Myocardial Injury

**Figure 4:**
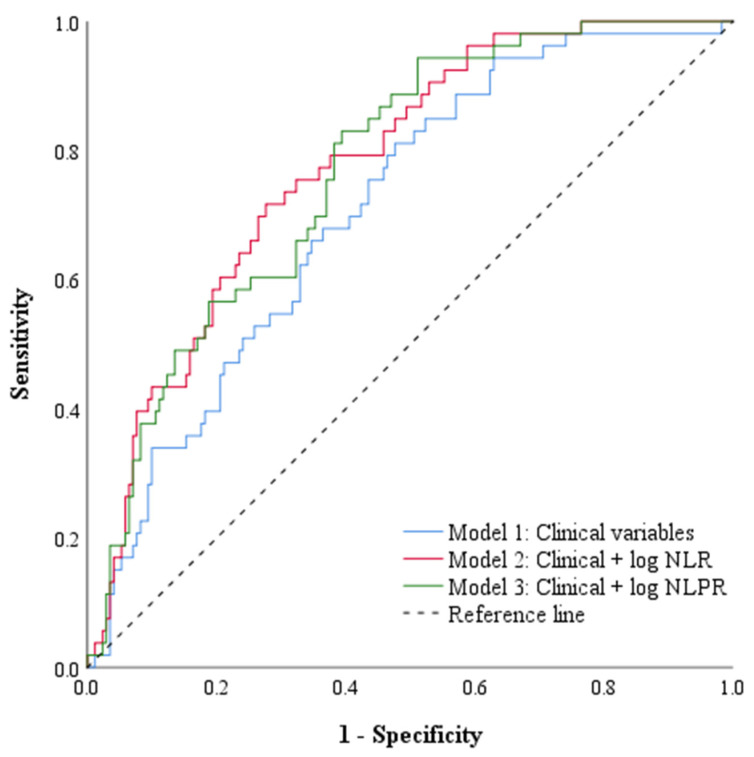
Receiver operating characteristic (ROC) curves for predicting high myocardial injury at presentation. ROC curves comparing discriminative performance of Model 1 (clinical variables only), Model 2 (clinical variables + log NLR), and Model 3 (clinical variables + log NLPR).

ROC analysis demonstrated numerically improved discriminative performance after incorporation of inflammatory indices into the baseline clinical model (Table 6 and Figure 4). The AUC increased from 0.709 for Model 1 to 0.778 after addition of log NLR and to 0.770 after addition of log NLPR. However, differences in AUC compared with Model 1 did not reach statistical significance by DeLong’s test (*p* = 0.058 and *p* = 0.087, respectively).

Additional reclassification analyses demonstrated improved classification performance after incorporation of inflammatory indices. Compared with Model 1, addition of log NLR demonstrated a continuous NRI of 0.679 and an IDI of 0.075. Similarly, addition of log NLPR demonstrated a continuous NRI of 0.429 and an IDI of 0.067.

## 4. Discussion

### 4.1. Principal Findings

In the present study, both log NLR and log NLPR were significantly associated with myocardial injury severity at presentation. These indices demonstrated moderate correlations with hs-Troponin T and remained independently associated after adjustment for conventional clinical variables and AMI subtype. Although improvements in ROC-based discrimination were modest, incorporation of inflammatory indices demonstrated additional explanatory and reclassification value for identifying high myocardial injury.

Among the evaluated indices, log NLR demonstrated slightly stronger associations, explanatory value, and discriminative performance across multiple analyses. Importantly, these associations remained significant after adjustment for AMI subtype, suggesting that inflammatory indices may reflect myocardial injury severity beyond differences attributable to AMI subtype alone. Collectively, these findings support the potential role of CBC-derived inflammatory indices as complementary markers associated with inflammatory responses during early AMI presentation.

While prior studies have primarily evaluated inflammatory indices in relation to diagnostic classification or clinical outcomes, the present study specifically focused on presentation-time myocardial injury severity using continuous hs-Troponin T levels. This approach provides additional insight into the relationship between systemic inflammation and the extent of myocardial injury in AMI.

### 4.2. Interpretation of Findings

#### 4.2.1. Baseline Characteristics

The baseline characteristics indicate that the study population consisted predominantly of older male patients with a moderate prevalence of traditional cardiovascular risk factors, including hypertension, smoking, dyslipidemia, and diabetes mellitus. This profile is consistent with typical populations presenting with suspected AMI [4,28,29].

Inflammatory markers were significantly elevated, as reflected by increased neutrophil counts, elevated NLR, and high NLPR values. These findings suggest the presence of a systemic inflammatory response at presentation, which is consistent with the established pathophysiology of AMI and myocardial injury. Elevated CBC-derived inflammatory indices in this cohort support the biological plausibility of evaluating CBC-derived biomarkers, particularly NLR and NLPR, in relation to myocardial injury severity.

Additionally, the markedly elevated hs-Troponin T levels and wide interquartile range suggest heterogeneous degrees of myocardial injury at presentation. This variability reinforces the clinical relevance of identifying additional biomarkers that may provide complementary information for assessing myocardial injury severity.

#### 4.2.2. Correlation Between Inflammatory Indices and Myocardial Injury

The observed positive correlations between inflammatory indices and hs-Troponin T support the role of systemic inflammation in myocardial injury. Both log NLR and log NLPR demonstrated moderate associations with hs-Troponin T, with log NLR showing slightly stronger performance across analyses. These findings are consistent with prior evidence demonstrating a positive association between NLR and myocardial injury. In a retrospective study of patients who underwent PCI, NLR was significantly positively correlated with CK-MB levels measured from coronary artery samples obtained before PCI, reflecting myocardial injury during the early phase. This temporal alignment supports the relevance of systemic inflammatory responses in early myocardial injury and is consistent with our findings using hs-Troponin T at presentation as a contemporary marker of myocardial injury severity [19].

The moderate R^2^ values observed in the scatter plots (0.180 for log NLR and 0.160 for log NLPR) indicate that inflammatory indices explain only part of the variability in hs-Troponin T levels. This finding is expected because myocardial injury in AMI is multifactorial and influenced by several clinical and pathophysiological factors beyond systemic inflammation alone [4,28,29].

Importantly, the consistency between scatter plot findings and Spearman correlation coefficients supports the robustness of the association between inflammatory indices and hs-Troponin T levels.

#### 4.2.3. Multivariable Regression Analysis

Multivariable regression analysis demonstrated that CBC-derived inflammatory indices, particularly log NLR and log NLPR, were independently associated with log-transformed hs-Troponin T after adjustment for conventional clinical variables and AMI subtype. Because STEMI and NSTEMI differ substantially in infarct burden, Troponin kinetics, and inflammatory activation, the persistence of these associations after adjustment for AMI subtype suggests that inflammatory indices may reflect myocardial injury severity beyond differences attributable to AMI subtype alone. Consistent findings from incremental model analyses further support the additional explanatory contribution of inflammatory indices beyond conventional clinical variables and AMI subtype.

Notably, conventional demographic or clinical variables were not independently associated with hs-Troponin T levels in the adjusted model. These findings may support the contribution of systemic inflammatory activation to myocardial injury severity during AMI presentation. Among the evaluated indices, log NLR demonstrated slightly stronger regression coefficients than log NLPR, suggesting relatively stronger associations with myocardial injury severity. Overall, the independent association observed for inflammatory indices support their potential role as early and readily available complementary biomarkers during AMI presentation.

#### 4.2.4. Incremental Association of Inflammatory Indices

The present study demonstrated that inflammatory indices provided one additional explanatory value beyond traditional clinical variables and AMI subtype. The baseline clinical model showed modest explanatory performance (R^2^ = 0.105), suggesting that conventional clinical variables and AMI subtype alone did not fully account for variability in myocardial injury severity at presentation. The addition of log NLR or log NLPR significantly improved model performance. Specifically, the addition of log NLR resulted in a significant increase in explanatory power (ΔR^2^ = 0.137), while log NLPR also significantly improved model performance (ΔR^2^ = 0.121).

These findings suggest that systemic inflammatory activation may provide additional information regarding myocardial injury severity beyond conventional clinical characteristics and AMI subtype classification. The slightly greater incremental contribution observed with log NLR further supports its potential role as a complementary inflammatory marker associated with myocardial injury during early AMI presentation.

#### 4.2.5. Multivariable Logistic Regression for High Myocardial Injury

Multivariable logistic regression analysis demonstrated that both log NLR and log NLPR were independently associated with high myocardial injury after adjustment for conventional clinical variables and AMI subtype, with log NLR showing a slightly higher odds ratio (OR −2.77 vs. 2.46). In addition, AMI subtype remained independently associated with high myocardial injury across all models. Because STEMI and NSTEMI differ substantially in infarct burden and inflammatory activation, these findings suggest that inflammatory indices may provide complementary information regarding myocardial injury severity beyond AMI subtype classification alone.

In contrast, conventional demographic and clinical variables were not independently associated with high myocardial injury in the adjusted models. These findings further support the association between systemic inflammatory activation and myocardial injury severity and suggest that inflammatory indices provide additional independent information beyond AMI subtype classification alone during early AMI presentation.

#### 4.2.6. Discriminative Performance for High Myocardial Injury

ROC analysis demonstrated modest improvement in discriminative performance after incorporation of inflammatory indices into the baseline clinical model. Although differences in AUC compared with the baseline model did not reach statistical significance after adjustment for AMI subtype, supplementary reclassification analyses demonstrated improved classification performance, particularly for log NLR. These findings suggest that inflammatory indices may provide additional clinically relevant information beyond conventional clinical variables and AMI subtype, even when improvements in global discrimination metrics are relatively modest.

### 4.3. Integrated Interpretation of Findings

Multiple complementary analytical approaches consistently demonstrated associations between CBC-derived inflammatory indices and myocardial injury severity at presentation. Across correlation, multivariable regression, incremental model, logistic regression, and ROC analyses, log NLR generally demonstrated slightly stronger associations and discriminative performance than log NLPR. These findings are consistent with the pathophysiological role of neutrophil activation and lymphocyte suppression in acute inflammatory responses contributing to plaque instability, thrombus formation, and myocardial injury [8,9,10,11,19,30].

The distinction between myocardial injury and myocardial infarction emphasized in the Fourth Universal Definition of Myocardial Infarction further supports the interpretation of the present findings. Because all patients had confirmed AMI, inflammatory indices were evaluated in relation to myocardial injury severity rather than diagnostic classification. Therefore, the observed associations with hs-Troponin T suggest that systemic inflammatory activation may be related to the extent of myocardial injury during early AMI presentation.

## 5. Conclusions

CBC-derived inflammatory indices, particularly log NLR and log NLPR, were independently associated with myocardial injury severity at presentation in patients with AMI, even after adjustment for AMI subtype. Although improvements in ROC-based discrimination were modest, supplementary reclassification analyses suggested incremental classification value beyond conventional clinical variables and AMI subtype. Overall, these findings support the potential role of CBC-derived inflammatory indices as accessible complementary biomarkers reflecting inflammatory responses associated with myocardial injury during early AMI presentation.

## 6. Strengths and Limitations

This study has several strengths. First, multiple complementary statistical approaches were employed, including correlation analysis, multivariable linear regression, incremental model evaluation, logistic regression, and ROC analysis, providing consistency findings across different analytical frameworks. Second, myocardial injury severity was evaluated using hs-Troponin T levels at presentation analyzed as both a continuous variable and tertile-based categories, allowing assessment across different levels of myocardial injury. Third, this study directly compared CBC-derived inflammatory indices, particularly NLR and NLPR, within the same cohort of patients with AMI. Among the evaluated indices, log NLR consistently demonstrated slightly stronger association and discriminative performance than log NLR, across multiple analyses. Finally, the evaluated inflammatory indices are derived from routine CBC testing, which is widely available in clinical practice and may support their potential applicability as accessible adjunctive biomarkers during early AMI presentation.

This study also has several limitations. First, due to the retrospective design, some clinical and laboratory variables were not uniformly available. In particular, eGFR was not consistently recorded, and cases with missing values were excluded from multivariable analyses. However, the number of missing values was small and is unlikely to have substantially influenced the results. Second, the relatively small sample size may limit statistical power and the precision of effect estimates. Third, time from chest pain onset was not incorporated into the analyses because of substantial missing data (69/252 cases, 27.4%) and potential inaccuracy in patient-reported symptom onset. Therefore, the observed associations may partly reflect differences in presentation timing rather than myocardial injury severity alone. In addition, several AMI severity-related variables were not included into the primary multivariable models because of incomplete availability and concerns regarding model stability relative to the available sample size. Hence, residual confounding related to symptom timing and infarct severity cannot be fully excluded. Finally, the present findings were derived from a retrospective single-center cohort, and internal and external validation were not performed. In addition, the same dataset was used for both model development and performance evaluation without internal validation techniques such as bootstrapping or cross-validation, which may have resulted in optimistic estimates of discriminative performance.

## 7. Implications for Clinical Practice and Future Research

Given its wide availability, low cost, and reproducibility, and rapid availability from routine laboratory testing, CBC-derived inflammatory indices, particularly NLR, may serve as a practical adjunct biomarker for early assessment of myocardial injury in patients with AMI. The observed incremental and reclassification value beyond conventional clinical variables and AMI subtype suggests that inflammatory indices may provide complementary information regarding myocardial injury severity at presentation and warrant further investigation for cardiovascular risk stratification and clinical decision support.

However, the modest explanatory and discriminative performance observed in this study indicate that CBC-derived inflammatory indices reflect only part of the multifactorial pathophysiology myocardial injury in AMI. Therefore, these inflammatory indices should be interpreted in conjunction with other clinical and laboratory parameters rather than as standalone biomarkers. Future studies incorporating additional clinical, imaging, and biochemical variables may further improve discriminative performance and enhance risk stratification models.

In addition, prospective multicenter studies with independent validation cohorts are warranted to further evaluate the robustness, generalizability, and clinical applicability of these findings. Longitudinal studies evaluating CBC-derived inflammatory indices in relation to long-term cardiovascular outcomes after AMI, particularly in chronic post-AMI care and cardiac rehabilitation may further clarify the role of persistent inflammatory activation in post-AMI remodeling, cardiovascular recovery, and long-term outcomes.

## Figures and Tables

**Table 1:** Baseline demographic, clinical, and laboratory characteristics of patients with acute myocardial infarction at presentation (0 h).

Variable	Overall (*n* = 252)
Demographic and clinical characteristics	
Age (years), median (IQR)	64 (15)
Male sex, *n* (%)	184 (73)
Smoking status, *n* (%)	99 (39.3)
Hypertension, *n* (%)	100 (39.7)
Diabetes mellitus, *n* (%)	54 (21.4)
Dyslipidemia, *n* (%)	71 (28.2)
Body mass index (BMI), kg/m^2^, Mean ± SD	23.44 ± 4.05
eGFR, mL/min/1.73 m^2^, mean ± SD	78.19 ± 24.24
Diagnosis	
STEMI	195 (77.4)
NSTEMI	57 (22.6)
Laboratory findings	
hs-Troponin T, ng/L, median (IQR)	416.00 (1718.75)
Neutrophil count (×10^12^ cells/m^3^)	7568 (5561)
Lymphocyte count (×10^12^ cells/m^3^)	1638 (1160)
Platelet count (×10^12^ cells/m^3^), median (IQR)	242.00 (89.50)
NLR, median (IQR)	4.47 (5.13)
NLPR, median (IQR)	1074.00 (1210.00)

Data are presented as median (interquartile range [IQR]), mean ± standard deviation (SD), or number (percentage), as appropriate. NLPR was calculated using neutrophil, lymphocyte, and platelet counts expressed as ×10^12^ cells/m^3^ without additional scaling. Abbreviations: BMI, body mass index; eGFR, estimated glomerular filtration rate; NSTEMI, non-ST-segment elevation myocardial infarction; STEMI, ST-segment elevation myocardial infarction; hs-Troponin T, high-sensitivity Troponin T; NLR, neutrophil-to-lymphocyte ratio; NLPR, neutrophil-to-lymphocyte–platelet ratio; IQR, interquartile range.

**Table 2:** Correlation between log-transformed inflammatory indices and log-transformed hs-Troponin T at presentation (0 h).

Variable	Spearman Correlation Coefficient (ρ)	*p*-Value
log NLR	0.422	<0.001 *
log NLPR	0.396	<0.001 *

Spearman correlation coefficients (ρ) were calculated using log-transformed inflammatory indices and log-transformed hs-Troponin T levels, due to non-normal distributions. All *p*-values are two-tailed. * *p* < 0.05. Abbreviations: NLR, neutrophil-to-lymphocyte ratio; NLPR, neutrophil-to-lymphocyte–platelet ratio; hs-Troponin T, high-sensitivity Troponin T.

**Table 3:** Multivariable linear regression models evaluating associations of clinical variables and inflammatory indices with log-transformed hs-Troponin T at presentation.

Variable	Model 1: Clinical Variables		Model 2: Clinical + Log NLR		Model 3: Clinical + Log NLPR	
	B Coefficient (95% CI)	*p*-Value	B Coefficient (95% CI)	*p*-Value	B Coefficient (95% CI)	*p*-Value
Intercept	5.93 (3.71 to 8.15)	<0.001 *	3.99 (1.85 to 6.14)	<0.001 *	−0.46 (−3.46 to 2.53)	0.761
Sex	0.28 (−0.34 to 0.89)	0.379	0.19 (−0.38 to 0.76)	0.515	0.24 (−0.34 to 0.81)	0.411
Age	−0.01 (−0.03 to 0.01)	0.359	−0.00 (−0.02 to 0.02)	0.749	0.00 (−0.02 to 0.02)	0.883
Smoking	−0.03 (−0.35 to 0.29)	0.847	0.04 (−0.26 to 0.33)	0.801	0.03 (−0.27 to 0.33)	0.834
Diabetes mellitus	−0.04 (−0.68 to 0.60)	0.902	0.06 (−0.54 to 0.66)	0.839	0.05 (−0.56 to 0.65)	0.883
Hypertension	−0.13 (−0.73 to 0.48)	0.681	−0.30 (−0.86 to 0.26)	0.292	−0.26 (−0.82 to 0.31)	0.375
Dyslipidemia	−0.07 (−0.67 to 0.53)	0.819	0.32 (−0.26 to 0.89)	0.276	0.22 (−0.36 to 0.79)	0.452
eGFR	−0.00 (−0.02 to 0.01)	0.479	−0.00 (−0.01 to 0.01)	0.856	−0.00 (−0.01 to 0.01)	0.906
AMI subtype	1.23 (0.64 to 1.82)	<0.001 *	1.01 (0.46 to 1.57)	<0.001 *	1.08 (0.52 to 1.64)	<0.001 *
log NLR	—	—	0.88 (0.60 to 1.16)	<0.001 *	—	—
log NLPR	—	—	—	—	0.77 (0.51 to 1.03)	<0.001 *

Multivariable linear regression analysis was performed using log-transformed hs-Troponin T as the dependent variable. Model 1 included clinical variables (sex, age, smoking status, diabetes mellitus, hypertension, dyslipidemia, eGFR, and AMI subtype) only. Model 2 included clinical variables plus log-transformed neutrophil-to-lymphocyte ratio (log NLR). Model 3 included clinical variables plus log-transformed neutrophil-to-lymphocyte × platelet ratio (log NLPR). * *p* < 0.05. Abbreviations: CI, confidence interval; eGFR, estimated glomerular filtration rate; NLR, neutrophil-to-lymphocyte ratio; NLPR, neutrophil-to-lymphocyte–platelet ratio.

**Table 4:** Incremental association of inflammatory indices with log-transformed hs-Troponin T at presentation.

Model	R	R^2^	ΔR^2^	*p*-Value
Model 1: Clinical variables	0.324 ^a^	0.105	–	–
Model 2: Clinical + log NLR	0.492 ^b^	0.242	0.137	<0.001 *
Model 3: Clinical + log NLPR	0.476 ^c^	0.226	0.121	<0.001 *

^a^ Model 1 included clinical variables (sex, age, smoking status, diabetes mellitus, hypertension, dyslipidemia, eGFR, and AMI subtype). ^b^ Model 2 included Model 1 variables plus log-transformed neutrophil-to-lymphocyte ratio (log NLR). ^c^ Model 3 included Model 1 variables plus log-transformed neutrophil-to-lymphocyte × platelet ratio (log NLPR). *p*-values were derived from the F-change test. * *p* < 0.05. Abbreviations: R, multiple correlation coefficient; R^2^, coefficient of determination; ΔR^2^, change in R^2^ compared with Model 1.

**Table 5:** Multivariable logistic regression analysis for high myocardial injury defined by the highest tertile of hs-Troponin T at presentation.

Variable	Model 1: Clinical Variables		Model 2: Clinical + Log NLR		Model 3: Clinical + Log NLPR	
	Adjusted OR (95% CI)	*p*-Value	Adjusted OR (95% CI)	*p*-Value	Adjusted OR (95% CI)	*p*-Value
Male sex	2.39 (0.92 to 6.21)	0.074	2.16 (0.79 to 5.88)	0.132	2.25 (0.82 to 6.13)	0.114
Age (per year)	0.99 (0.96 to 1.02)	0.398	0.99 (0.96 to 1.02)	0.612	1.00 (0.97 to 1.03)	0.882
Smoking	0.77 (0.49 to 1.19)	0.238	0.83 (0.53 to 1.32)	0.439	0.84 (0.53 to 1.33)	0.449
Diabetes mellitus	1.22 (0.46 to 3.21)	0.689	1.46 (0.52 to 4.10)	0.469	1.43 (0.51 to 3.97)	0.498
Hypertension	0.97 (0.40 to 2.35)	0.942	0.79 (0.31 to 1.99)	0.614	0.87 (0.35 to 2.18)	0.773
Dyslipidemia	0.72 (0.28 to 1.83)	0.492	1.10 (0.41 to 2.99)	0.846	0.96 (0.36 to 2.57)	0.942
eGFR	0.99 (0.98 to 1.01)	0.264	0.99 (0.98 to 1.01)	0.326	0.99 (0.98 to 1.01)	0.334
AMI subtype	16.99 (2.24 to 129.16)	0.006 *	15.44 (1.88 to 127.13)	0.011 *	16.93 (2.08 to 138.01)	0.008 *
log NLR	—	—	2.77 (1.65 to 4.66)	<0.001 *	—	—
log NLPR	—	—	—	—	2.46 (1.53 to 3.95)	<0.001 *

Multivariable logistic regression analysis was performed to evaluate factors associated with high myocardial injury, defined as the highest tertile of hs-Troponin T. Model 1 included clinical variables (sex, age, smoking status, diabetes mellitus, hypertension, dyslipidemia, eGFR, and AMI subtype). Model 2 included Model 1 variables plus log-transformed neutrophil-to-lymphocyte ratio (log NLR). Model 3 included Model 1 variables plus log-transformed neutrophil-to-lymphocyte–platelet × platelet ratio (log NLPR). * *p* < 0.05. Abbreviations: OR, odds ratio; CI, confidence interval; eGFR, estimated glomerular filtration rate; NLR, neutrophil-to-lymphocyte ratio; NLPR, neutrophil-to-lymphocyte–platelet ratio.

**Table 6:** Discriminative and reclassification performance of models for high myocardial injury at presentation.

Model	AUC (95% CI)	ΔAUC	*p*-Value vs. Model 1	Continuous NRI	IDI
Model 1: Clinical variables	0.709 (0.635–0.783)	–	–	–	–
Model 2: Clinical + log NLR	0.778 (0.712–0.843)	+0.069	0.058	0.679	0.075
Model 3: Clinical + log NLPR	0.770 (0.704–0.836)	+0.061	0.087	0.429	0.067

Model 1 included clinical variables (sex, age, smoking status, diabetes mellitus, hypertension, dyslipidemia, eGFR, and AMI subtype). Model 2 included Model 1 variables plus log-transformed neutrophil-to-lymphocyte ratio (log NLR). Model 3 included Model 1 variables plus log-transformed neutrophil-to-lymphocyte × platelet ratio (log NLPR). *p*-values for ΔAUC were calculated using DeLong’s test for comparison of correlated AUCs versus Model 1. Continuous NRI and IDI were calculated relative to Model 1. Abbreviations: AUC, area under the receiver operating characteristic curve; CI, confidence interval; ΔAUC, difference in AUC compared with Model 1; eGFR, estimated glomerular filtration rate; IDI, integrated discrimination improvement; NLR, neutrophil-to-lymphocyte ratio; NLPR, neutrophil-to-lymphocyte–platelet ratio; NRI, net reclassification improvement.

## Data Availability

The raw data supporting the conclusions of this article will be made available by the authors on request.

## Abbreviations

The following abbreviations are used in this manuscript:

ACS	Acute coronary syndromes
AMI	Acute myocardial infarction
UA	Unstable angina
STEMI	ST-segment elevation myocardial infarction
NSTEMI	Non-ST-segment elevation myocardial infarction
PCI	Percutaneous coronary intervention
hs-Troponin T	High-sensitivity Troponin T
CBC	Complete blood count
WBC	White blood cell
NLR	Neutrophil-to-lymphocyte ratio
NLPR	Neutrophil-to-lymphocyte × platelet ratio
BMI	Body mass index
eGFR	estimated glomerular filtration rate
ROC curves	Receiver operating characteristic (ROC) curves
AUC	Area under the ROC curve
95% CI	95% confidence intervals
IDI	Integrated discrimination improvement
NRI	Net reclassification improvement
